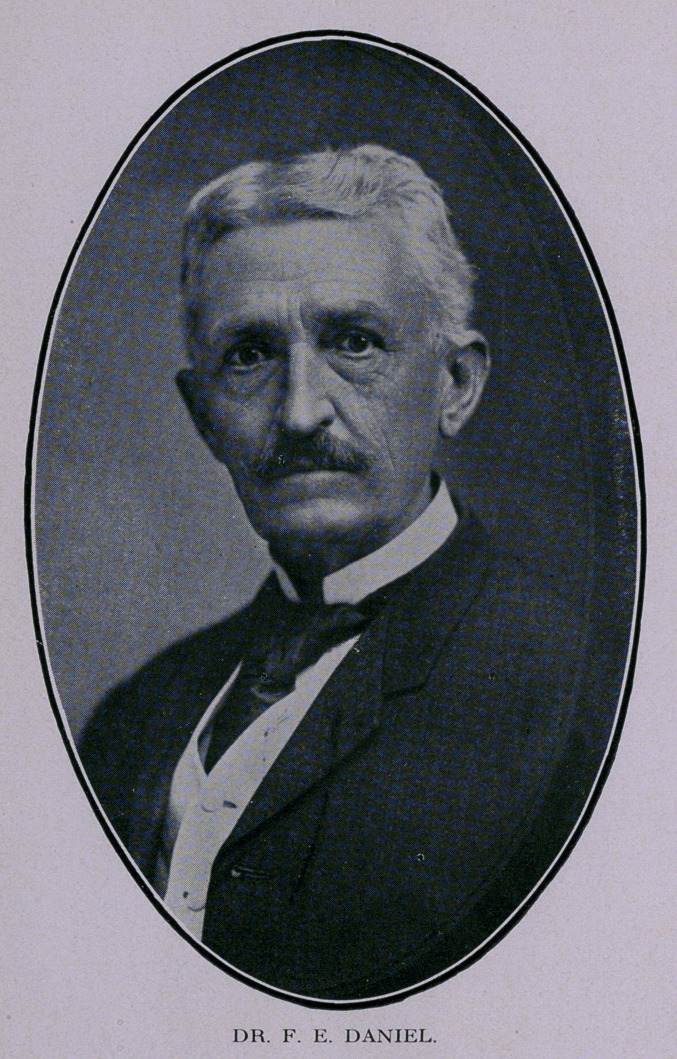# Dr. Grace’s Tribute

**Published:** 1914-06

**Authors:** 


DR. F. E. DANIEL.

THE

### TEXAS MEDICAL JOURNAL.

**Dr. F. E. Daniel, Founder. Established July, 1885**

MRS. F. E. DANIEL, - - - - - - - Publisher and Managing Editor

Published Monthly.—Subscription, $1.00 a Year.

VOL. XXIX. AUSTIN, JUNE, 1914 No. 12.

The publisher is not responsible for the views of the contributors.

### Dr. Grace's Tribute.

Surrounded by his loved ones, Dr. Ferdinand Eugene Daniel departed this life on May 14th, at 7:30 p. m. at his home, 204 East Tenth Street, Austin, Texas.

At the passing of this great and good man, all Texas physicians bow their heads in sorrow.

Dr. Daniel was by birth a Virginian, and was educated in Mississippi and Louisiana. In his early manhood he chose Texas for his field of labor. At the breaking out of the Civil War he joined Company K of the Eighteenth Mississippi Regiment. After serving for a time he resumed his medical course in New Orleans, and graduated in 1862.

In July, 1862, he received the appointment of surgeon in the Confederate service with the rank of major, and served until the close of the war. Many positions of honor and usefulness came to him during this service.

Having previously studied and acquired a knowledge of law, he was appointed Judge Advocate for the general court-martial of the army in Tennessee. He was secretary of the army board of medical examiners in General Bragg's army, and later active in the Kentucky campaign, being attached to the staff of General Hardee.

Dr. Daniel was richly endowed by nature. Erect and with a commanding presence, his superb stature towered above the host of his fellows. His was an eye whose keenness and sternness brought terror to the evil-doer, but that melted instantly into tenderness and sympathy for those in misfortune and distress. When to these Mother Nature added a mind whose brilliancy stood out with the brightest in the galaxy of medical stars and whose splendid accomplishments were no less brilliant in many other directions, it made of him indeed a leader of men.

Reared under the conditions of the Old South and nurtured in her institutions of learning, he retained, until his death, that noble dignity and courtesy of manner, that consideration and solicitude for the feelings and welfare of others, that love for his family, that profound respect for all womankind, that loyalty of friendship, that high standard of integrity and that moral and physical courage that typify the gentlemen of the Old South.

One of the foremost in State medicine and hygiene, his pen was at all times wielded in the service of his fellowman. As editor of his beloved TEXAS MEDICAL JOURNAL, or "Red Back," as it is more familiarly called, his forceful, bright, snappy editorials, as well as his writings in a deeper vein, served to place the journal in the front rank, and probably made of it the most popular of Southern medical journals.

As a surgeon in the Confederate service in the Civil War, Dr. Daniel served with distinction not only in ministering to the sick and wounded, but his presence was a constant encouragement to his fellows. The kindly bearing and constant good humor and consideration for those about him soothed and cheered the hard life of the ragged and starving soldier.

His "Recollections of a Rebel Surgeon," a masterpiece of anecdote, sparkling with wit and repartee, was taken largely from his experiences during this troublous time. Splendid man! gleaning and disseminating the wine of hope, gathering and scattering rays of sunshine while the black and gloomy war clouds hung so heavily over his beloved Southland.

As a monument to the scientific side of this great nature stands that splendid work, "The Strange Case of Dr. Bruno." The sting of the mud-wasp producing a state of suspended animation on its prey serves as the basis around which is woven a story that rivals the best of the productions of Edgar Allan Poe or Sir Conan Doyle.

As an orator and after-dinner speaker, Dr. Daniel had but few equals. A splendid command of English, the sparkling wit of a Mark Twain, coupled with the soul-touching pathos of Bob Taylor, enabled him to sway his audiences at will. The writer has seen this accomplished speaker lead his audience from laughter to tears by his beautiful word-pictures placed before his auditors in simple easy manner.

In 1866 Dr. Daniel located in Galveston, where he was one of the founders and teachers in the first Texas Medical College. His constant labors for, and loyalty to, ethical medical organization through his JOURNAL, in the councils of the Texas Medical

Association, and wherever word or work was needed, justly entitled him to the sobriquet, "the Father of Medicine" in Texas, and as a reward he was honored by the highest gift within the power of the State Medical Association of Texas—that of the Presidency in 1905. It seems a singular coincidence that at 7:30 on the evening of May 14th, when the last echoes of this past session of the great Medical Association that he loved so much were dying away his soul took its flight to the eternal shores.

In 1906 Dr. Daniel was honored by election to the Presidency of the International Congress on Tuberculosis, which held its session during his incumbency in New York City.

His was a fine Christian character, fearless in combat, always found on the firing line, or, if need be, alone, fighting for what he believed to be a righteous cause, and never shirking a duty. He was honorable and upright in character, loving his fellowman, loyal in friendship and just toward all in the sight of God and man.

Physician, author, orator, soldier, statesman and beloved friend! No longer in the halls of deliberation and counsel will we be able to turn to thee for advice; no longer will that voice be raised around the boards in encouragement to thy fellow physicians; no longer will we grasp that hand and feel its cordial welcoming pressure at our annual gatherings. Thou hast left us a life worthy of emulation, example fruitful of good and fraught with happiness and good-will to all mankind. That noble spirit is with God, and we ask that, if possible, it come to us in our dark and weary hours and serve to light us and lead us out of the depths. We loved thee, all Texas loved thee, and this well-merited heritage, the love of thy fellowman, will stand as a monument to glorify thy memory. May God rest thy soul in peace!

M. B. GRACE.

---

CORPUS CHRISTI, TEXAS, Box 639, June 1, 1914.

*Mrs. Josephine E. Daniel, Austin, Texas.*

DEAR MRS. DANIEL: It is impossible for me to write you just how I feel over the death of your lamented husband, my old and much beloved friend, who was so much to me during the last thirty years of his honorable and well spent life.

Although "native to the manor born"—Old Virginia—and had known of each other for a number of years, we never met until 1882, when a mutual friendship sprang up between us that ripened into deep and a brotherly affection, which I know he retains for me in the spirit, as I do, and ever shall, for him, in this, our material world.

To me, Dr. Daniel was typical of that generation now well-nigh extinct,—a polished old Virginia gentleman; consequently, he was courteous, upright, manly, generous, just, honorable, hospitable, tender—as a child he worshipped little children—and as fearless as the bravest. He embodied all that is good, and noble, and true in man, and in his going the crown-jewel in the galaxy of my nearest and dearest friends went with him forever.

Those who knew Dr. Daniel best loved him most, and, being of those who knew him well, I am one of the many who mourn his death as a loss, personally to his profession—which he adorned as is permitted to but few—and to humanity at large, which is irreparable.

"Peace to his ashes; eternal rest to his soul."

With renewed assurances of my profoundest sympathy and of highest esteem, I am, dear madam,

Yours very sincerely,

R. H. L. Bibb.

## Figures and Tables

**Figure f1:**